# Health of Louisville

**Published:** 1853-10

**Authors:** 


### THE WESTERN JOURNAL.

Third Series.—Vol. XII.—No. IV.

LOUISVILLE, OCTOBER 1, 1853.

### HEALTH OF LOUISVILLE.

Having begun to notice the unusual prevalence of health in Louisville during the season which is ordinarily the sickly one, it affords us pleasure to be able to continue our favorable report. As we have had occasion already to remark, notwithstanding the profusion of fruits which the season has afforded, the population of our city have enjoyed a most remarkable exemption from all complaints of the bowels. The experiment has been tried on the largest scale, and it is impossible that the result could have been more decisive. After the experience of the past summer it would be folly in a Louisville physician ever to connect summer complaints with summer fruits. Autumn has brought no unfavorable change. We have not for many years had as few cases of fever in September as the last month has produced. The season has been altogether a remarkable one — remarkable, in the early part, for the drouth, and remarkable throughout for its heat, and for its healthfulness and its fruitfulness.

